# The Occurrence of Genetic Alterations during the Progression of Breast Carcinoma

**DOI:** 10.1155/2016/5237827

**Published:** 2016-04-14

**Authors:** Xiao-Chen Li, Chenglin Liu, Tao Huang, Yang Zhong

**Affiliations:** ^1^School of Life Sciences, Fudan University, Shanghai 200433, China; ^2^School of Life Sciences and Biotechnology, Shanghai Jiaotong University, Shanghai 200240, China; ^3^Institute of Health Sciences, Shanghai Institutes for Biological Sciences, Chinese Academy of Sciences, Shanghai 200031, China

## Abstract

The interrelationship among genetic variations between the developing process of carcinoma and the order of occurrence has not been completely understood. Interpreting the mechanisms of copy number variation (CNV) is absolutely necessary for understanding the etiology of genetic disorders. Oncogenetic tree is a special phylogenetic tree inferential pictorial representation of oncogenesis. In our present study, we constructed oncogenetic tree to imitate the occurrence of genetic and cytogenetic alterations in human breast cancer. The oncogenetic tree model was built on CNV of ErbB2, AKT2, KRAS, PIK3CA, PTEN, and CCND1 genes in 963 cases of tumors with sequencing and CNA data of human breast cancer from TCGA. Results from the oncogenetic tree model indicate that ErbB2 copy number variation is the frequent early event of human breast cancer. The oncogenetic tree model based on the phylogenetic tree is a type of mathematical model that may eventually provide a better way to understand the process of oncogenesis.

## 1. Introduction

Copy number variations (CNVs) are a form of structural genetic alterations contributed to the initiation and progression of breast cancer. They are an underlying cause of the cancer cell population adaption by affecting cell evolution including proliferation, fitness, and clonal selection [1]. Distinct CNV patterns have been reported in breast cancer subgroups, which are correlated to different stages of cancer progression [2]. The study to understand the evolutionary relationship of CNVs based on their occurrence can reveal important biomarkers and benefit the diagnostic of breast carcinoma.

A powerful tool to reveal the evolutionary relationships is the phylogenetic tree. Besides its application to depict the evolutionary descent of different species or organisms from the same ancestor, it is widely used in cancer study to reveal the phylogenetic relationships of tumor subclones during cell evolution. Based on the assumption that each of the subclones derived from a parental cell population carries all its alterations and acquires additional ones, phylogenetic tree describes this linear cancer genome evolution by constructing an oncogenetic tree representing common sequences of genetic events in tumor progression. The oncogenetic models are flexible since they can represent multiple pathways at the same time [3]. Various bioinformatics software programs have been proposed for the construction of oncogenetic tree including TrAp [4] and oncotree [5].

In this paper, we construct two oncogenetic trees based on independent models based for human breast carcinoma. In the distance-based oncogenetic tree construction, the state of a normal cell is the root of the tree [6]. Each subclone is represented by an imbalanced tree, with the nodes being described by probabilities of CNV occurrence based on conjunctive Bayesian networks. CNV occurring early in the cytogenetic evolution event is close to the root in the tree. The gene positive selection is conveyed by degree of the imbalance or asymmetry of the tree topology, while those closely balanced subsets are known as clusters in the tree and hidden genetic events before the internal crossover point. However, the limitation is the insufficiency to explain the negative correlation between two aberrations. Hence, we verify our results by introducing the branching oncogenetic trees to estimate the probabilities of false negative and false positive observations [7].

## 2. Material and Methods

The sequence and CNA data of 963 breast cancer patients were collected from the Cancer Genome Atlas (TCGA) Breast Invasive Carcinoma project. Important copy number variations were selected by searching the key words “copy number alterations”, “CNAS”, “breast cancer”, “deletion”, and “amplification” in Online Mendelian Inheritance in Man (OMIM), and candidate alteration biomarkers for breast cancer were chosen as ErbB2, AKT2, KRAS, PIK3CA, PTEN, and CCND1, whose copy number alterations we supposed would play a key role in the development of breast cancer based upon literature research.

The distance-based oncogenetic tree model is constructed based on the idea of path models by Desper et al. [7]. The branches of the tree represent the multiple pathways of the given tumor. The pathways either are parallel to each other or branch out in tree topology, each showing a dependent or independent event. Based on first-order Markov property, a genetic event only depends on its single direct predecessors in the tree. The occurrence probability of the subclones derived from their parental clones is described as conditional probability and represented as the edge weight in the parent-child relations in the tree topology. In this tree model, the root of the tree serves as a normal event and the inner nodes represent the hypothetical taxonomic units. A probability *p* ^*e*^ is assigned to each edge *e* in the tree. In the beginning, none of events corresponding to the nodes in the tree has occurred. Starting at the root of the tree, the event of the next node occurs based on the model regulations using the following probabilities. Denote the occurring event as node *X* in the tree; it occurs independently in a continuous marginal probability *p* ^*e*^. In this tree model, the weights of edges are defined as −log⁡(*p* ^*e*^). Finally, as to a single tumor, the subset of leaf events occurring are the results of the random experiment. The clusters of trees characterize the subset of the changes that have close correlations [8], and clusters close to root reveal the early events. Maximum likelihood estimation is performed to determine the best fitting tree model for the given copy number data. The nonparametric bootstrap [9] is adopted to evaluate the uncertainty of properties of the estimated tree model. The bootstrap confidence values are obtained for the inner edges and the resulting tree displays the splits that occurred in >10 percent of the bootstrap datasets [10].

Two types of oncogenetic tree models were proposed to describe the occurrence of copy number alterations during the progression of breast cancer. The branching trees were constructed using the software Oncotree [5], (https://cran.r-project.org/web/packages/Oncotree/), while the distance-based trees were generated by the R software package (https://cran.r-project.org/web/packages/oncomodel/) [10].

## 3. Results

### 3.1. Frequency of Gene Alteration

**Figure 1:**
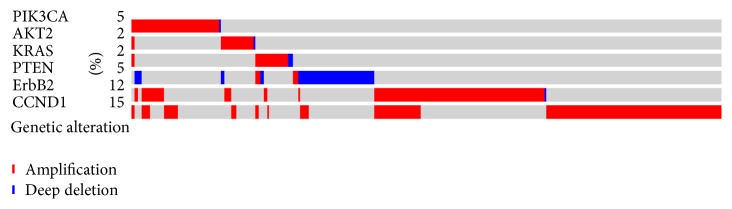
The CNV frequency of six candidate genes in the breast carcinoma patients. The CNVs of at least one of the six candidate genes occur in 342 (36%) of 963 cases/patients. The CNV frequencies of the six candidate genes are PIK3CA (5%, 51/963), AKT2 (2%, 22/963), KRAS (3%, 25/963), PTEN (6%, 55/963), ErbB2 (13%, 121/963), and CCND1 (16%, 153/963).

The copy number variation is detected in at least one of the six candidate genes in thirty-six percent (342 in 963 cases) of patients. Among the six copy number alterations assayed, the CCND1 alteration was detected as the most frequent one, existing in 16% of breast cancer cases. AKT2 alteration was the least common, found in only 2% of patients. The CNV frequencies of the other four candidate genes are PIK3CA (5%, 51/963), KRAS (3%, 25/963), PTEN (6%, 55/963), and ErbB2 (13%, 121/963), as shown in Figure 1.

**Figure 2:**
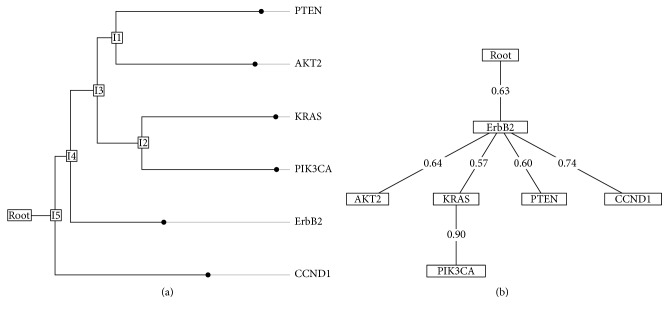
The distant-based and branching oncogenetic tree models for copy number alterations in 963 breast tumors. (a) In the distance-based oncogenetic tree, hidden or unknown events are represented as internal nodes. Leaves between the distances depend on the meaning of copy number changes with greater probability of cluster-related copy number variation. The root of the tree close to the left of the last point in the imbalance of genetic evolution occurred early. Interconnection path represents a high correlation between events, forming clusters of genetic balance. (b) In the branching oncogenetic tree, root represents the event with no alterations. The alterations genes include ErbB2, AKT2, KRAS, PIK3CA, PTEN, and CCND. The aberration of ErbB2 is potentially early event as it is close to the root and also the root of a new subtree.

According to the distance-based oncogenetic trees (Figure 2(a)), the branch of ErbB2 event yields two clusters, including KRAS and PIK3CA and AKT2 and PTEN. In this model, the interconnection of the paths forms the cluster of genetic imbalances which indicates high correlation of events. The closer the final points are to the root of the tree, the earlier the imbalances occur in genetic evolution. The ErbB2 alteration, presumably a primary event, is shown to be close to the root of the tree. The next branching in the tree separates CCND1 from the remaining events.

The branching oncogenetic tree model of the dataset showed four branches departing from the root (Figure 2(b)). ErbB2 was an important early event as it is close to the root and also the root of a new subtree. The CCND1 alteration, PTEN alteration, and AKT2 alteration are located on one branch emanating from the root. Those branches do not continue to any other alteration. The next main branch emanating from the root leads to alteration of ErbB2, which is marked as a first node, and then branches again to KRAS and PIK3CA alteration. The KRAS subbranch continued to its alteration to PIK3CA with high probability.

**Figure 3:**
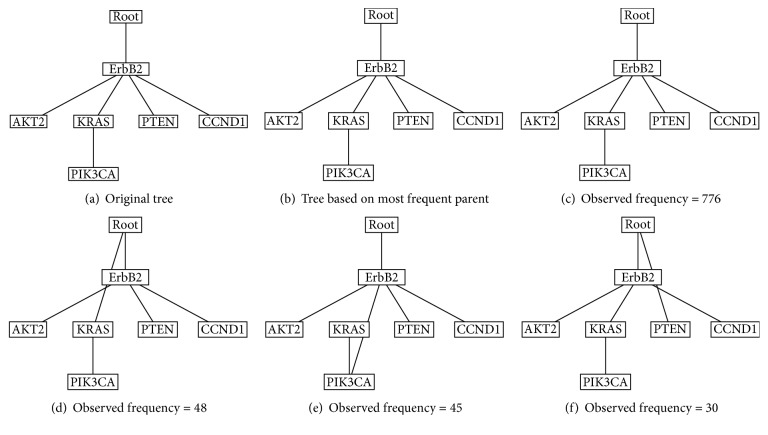
The frequent oncogenetic trees constructed in the 1000-time bootstrap resampling procedure for the primary *n* = 963 trees. (a) The original tree; (b) the tree based on the most frequent parent; (c) the tree repeatedly appearing 776 times; (d) the tree replicated 48 times; (e) the tree replicated 45 times; (f) the tree replicated 30 times.

In the 1000-time replicated bootstrap resampling procedure for the primary tree, the original tree is reconstructed in 776 times, which is the most frequent tree in the bootstrapping. The frequent trees and the original tree are showed in Figure 3. Among trees representing at least 1000 resampled oncogenetic trees, AKT2, KRAS, PTEN, and CCND1 are direct or indirect descendants of ErbB2. ErbB2 is always close to the root, and KRAS and PIK3CA always stay in one branch. In 48 cases in the bootstrapping, the KRAS-PIK3CA branch is independent of ErbB2 from the root. In another 45 cases, the PICKA subbranch consistently shows high probability of alteration to ErbB2 and then to KRAS. PTEN is an independent branch of the root in the tree in 30 cases.

## 4. Discussion

In this paper, we construct the distance-based tree models and the branching models based on six candidate genes whose CNVs are reported as important to breast carcinoma based on OMIM database. Based on these constructed oncogenetic tree models, some common findings are revealed as the following inferences: (a) ErbB2 is close to the root of the tree, indicating that its alteration is potentially an early event in breast cancers; (b) at least three subtrees appear to follow up the event of ErbB2, which indicates that there are pathways of progression in human breast cancer; (c) a close relationship is revealed between KRAS and PIK3CA alterations, since they are consistently grouped to the same subcluster.

Some contradictories are discovered to be mainly relative to the order of KRAS and PIK3CA alteration, that is, whether PIK3CA alteration as a part of RAS pathway is the late event after KRAS alteration or PIK3CA the late event in breast cancer. Hollestelle et al. reported that PIK3CA alteration occurs almost exclusively in invasive tumors, which is a late event of the PI3K pathway [11]. However, Hollestelle et al. found that there are downstream effectors of the oncogenic PI3K and RAS pathways in breast cancer [11]. However, as shown in our results, KRAS and PIK3CA alteration tend to occur after ErbB2, and a close relationship must exist between them.

According to the ErbB2 branch, copy number alterations of AKT2, PTEN, CCND1, KRAS, and PIK3CA may associate with the upstream pathway lesions on the ErbB2 pathway based on oncogenetic tree theory. It is reported that ErbB2 is a major driver of tumor growth in 20% of breast cancers [12]. The downstream signaling cascade PI3K signaling associated with signals originating from HER2 plays a vital role in tumorigenesis, drug resistance, and tumor progression in breast cancer [13]. CCND1 and ErbB2 have been reported to play an early role in sporadic breast cancer [14]. The link between ErbB2 and the AKT pathway in breast cancers has been validated in vitro and in vivo, indicating the possible role of AKT activation in ErbB2-mediated breast cancer progression [15].

Based on the oncogenetic tree model of human breast carcinoma, we demonstrate the following three key points:

- The alterations of ErbB2 are a potential early event in cellular transformation of breast cancer.
- The copy number alterations of AKT2, PTEN, CCND1, KRAS, and PIK3CA may associate with their upstream pathway lesions ErbB2 alterations.
- A close relationship exists between KRAS and PIK3CA alterations.

In summary, the oncogenetic tree models can provide a better way to understand the process of oncogenesis. They can be applied to other somatic alterations analysis to describe a reasonable order of biological events for the major pathways of cancer.
